# Supplementary material for: YAP charge patterning mediates signal integration through transcriptional co-condensates
Source: Nat Commun. 2025 Aug 12;16:7454. doi: 10.1038/s41467-025-62157-3 (PMC12343768; doi:10.1038/s41467-025-62157-3)
Supplement: Supplementary file 1 — Supplementary Information [file 41467_2025_62157_MOESM1_ESM.pdf]

Supplementary Figures

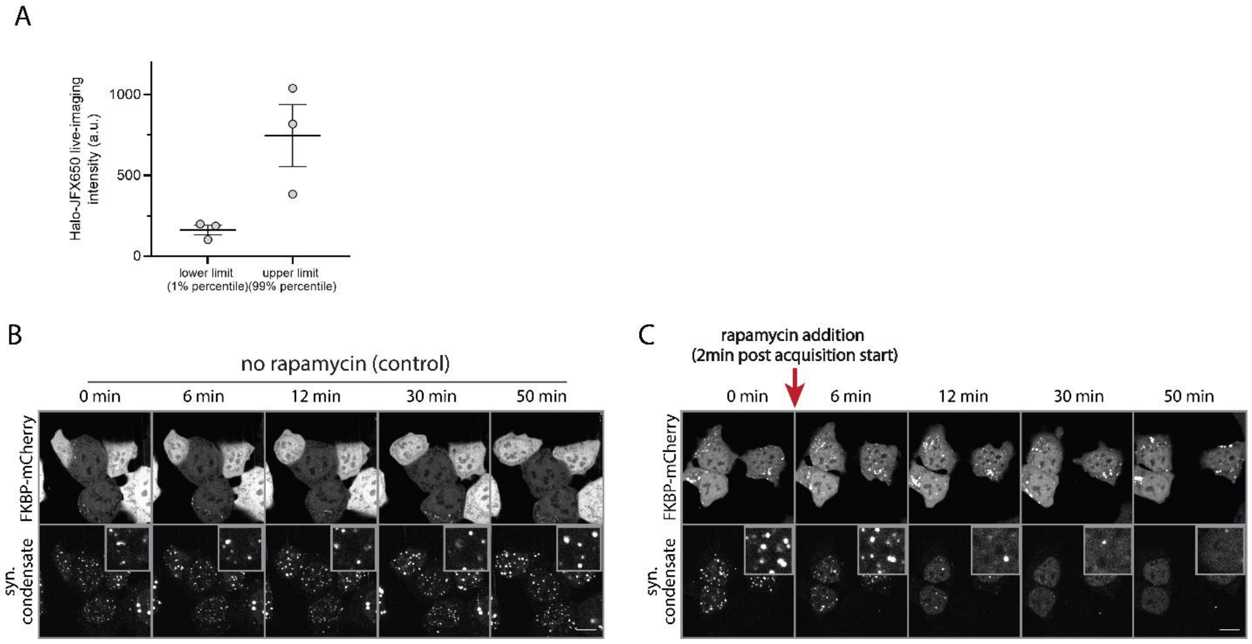

Figure S1 Verification of mCherry as solubility tag

A) Quantification of the endogenous YAP expression range (1-99% percentile) in the Halo-YAP (JFX650) re-expression system. The Halo-tag (JFX650) cells were IF stained for YAP and compared to IF stains of WT cells. Shown is the Halo-YAP (JFX650) intensity (y-axis) corresponding to the lower 1% percentile and upper 99% percentile of the WT distributions (x-axis). Shown are mean +/- SEM from N = 3 independent experiments. **B-C**) Verification of mCherry as a solubility tag in mESCs using the synthetic condensate system (SPARK-ON). Cells expressing the SPARK-ON components and FKBP-mCherry (top row). Pre-formed synthetic condensates (bottom row) were left untreated (B) or acutely treated with rapamycin at 2 min post-acquisition start (B). Note the dissolution of condensates upon mCherry recruitment (inset, bottom row, C) as compared to the control (inset, bottom row, B). Scale bar: 10 μm

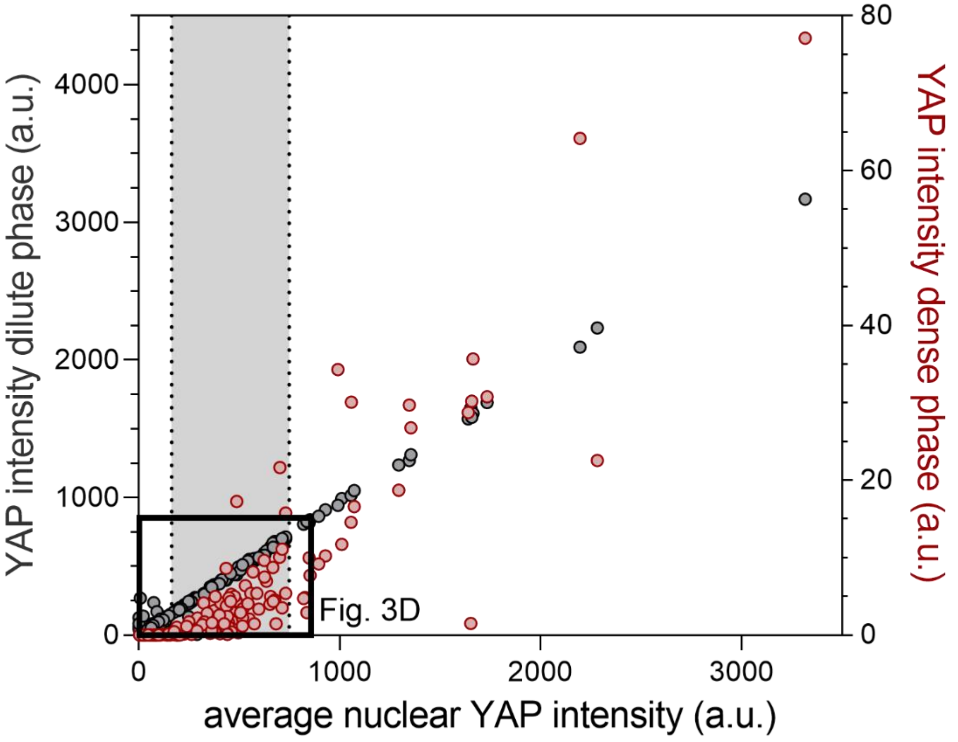

Figure S2 Extended dataset for dense and dilute phase YAP intensities

Full dataset of data shown in Fig. 3D (indicated by black rectangle). For details see Figure legend of Fig. 3D.

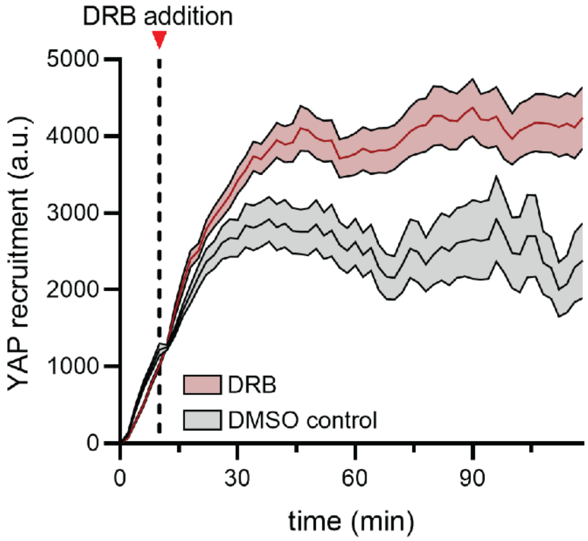

Figure S3 Quantification of YAP recruitment to synthetic condensates upon inhibition of RNA synthesis

Quantitation of YAP recruitment to synthetic condensates following inhibition of RNA synthesis with DRB at t = 12 min (dashed line). DMSO serves as the control. Shown are mean +/- SEM from pooled time courses of N = 4 independent experiments.

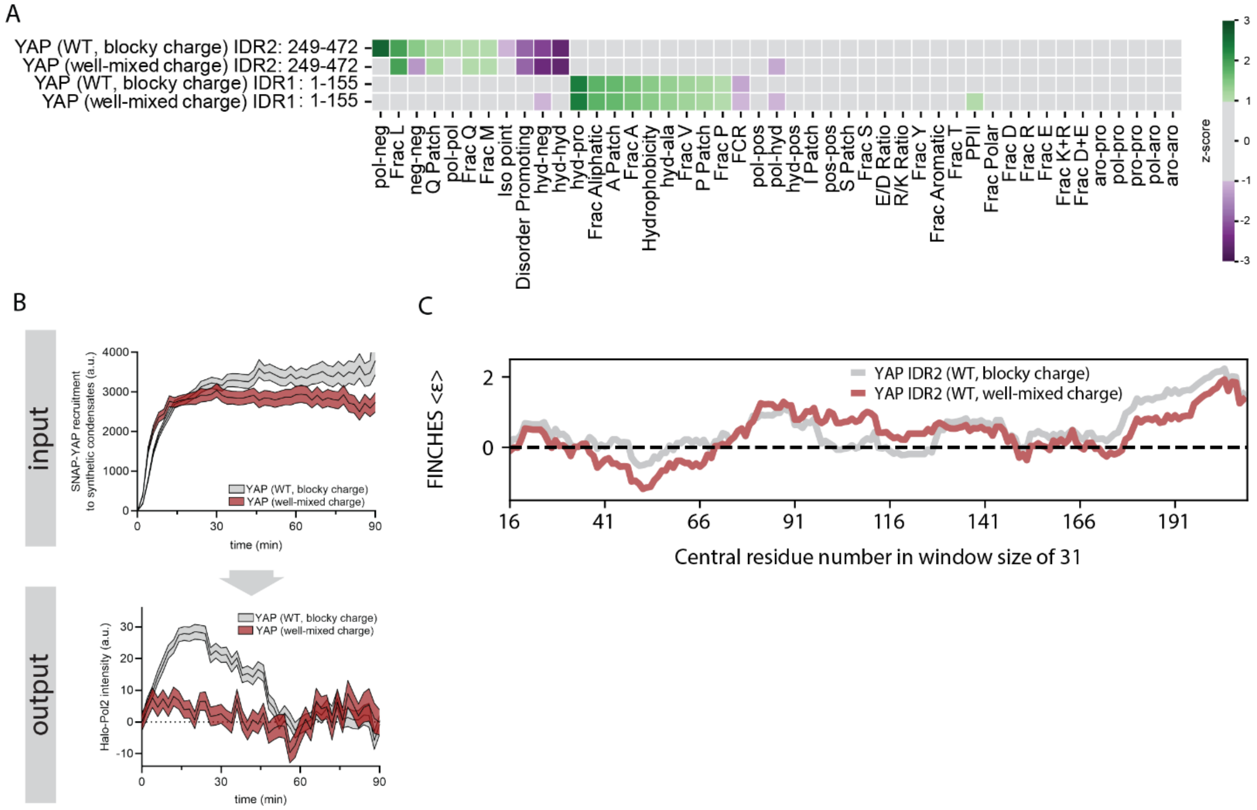

Figure S4 IDR grammar analysis for well-mixed charge YAP variant

A) IDR grammar analysis for sequence features across the YAP IDRs 1 and 2 for the WT and well-mixed charge variant. See IDR Grammar Key in Fig. 6B for features analyzed. **B)** Endogenous Pol2 recruitment to acute YAP condensate formation using the synthetic condensate system (see Fig. 4A), comparing the WT blocky-charge protein with the engineered well-mixed charge variant. Top (input): Recruitment of SNAP-YAP variants (WT blocky charge vs well-mixed charge) to synthetic condensates. Bottom (output): Endogenous Halo-Pol2 response to the acute YAP condensate recruitment (top graph). Shown are mean +/- SEM of pooled time series from N = 4 independent experiments. **C**) Predicted intermolecular interaction maps for the Pol2 IDR and YAP IDR2 of the WT (blocky charge; grey line) and engineered well-mixed charge YAP variant (red line). Epsilon (y-axis) represents the predicted attracting (negative epsilon) and repelling (positive epsilon) interaction based on a number of physio-chemical protein features. Note that the attractive interaction with Pol2 (negative epsilon for residues ~40-70) is predicted to be slightly enhanced in the well-mixed charge YAP variant.

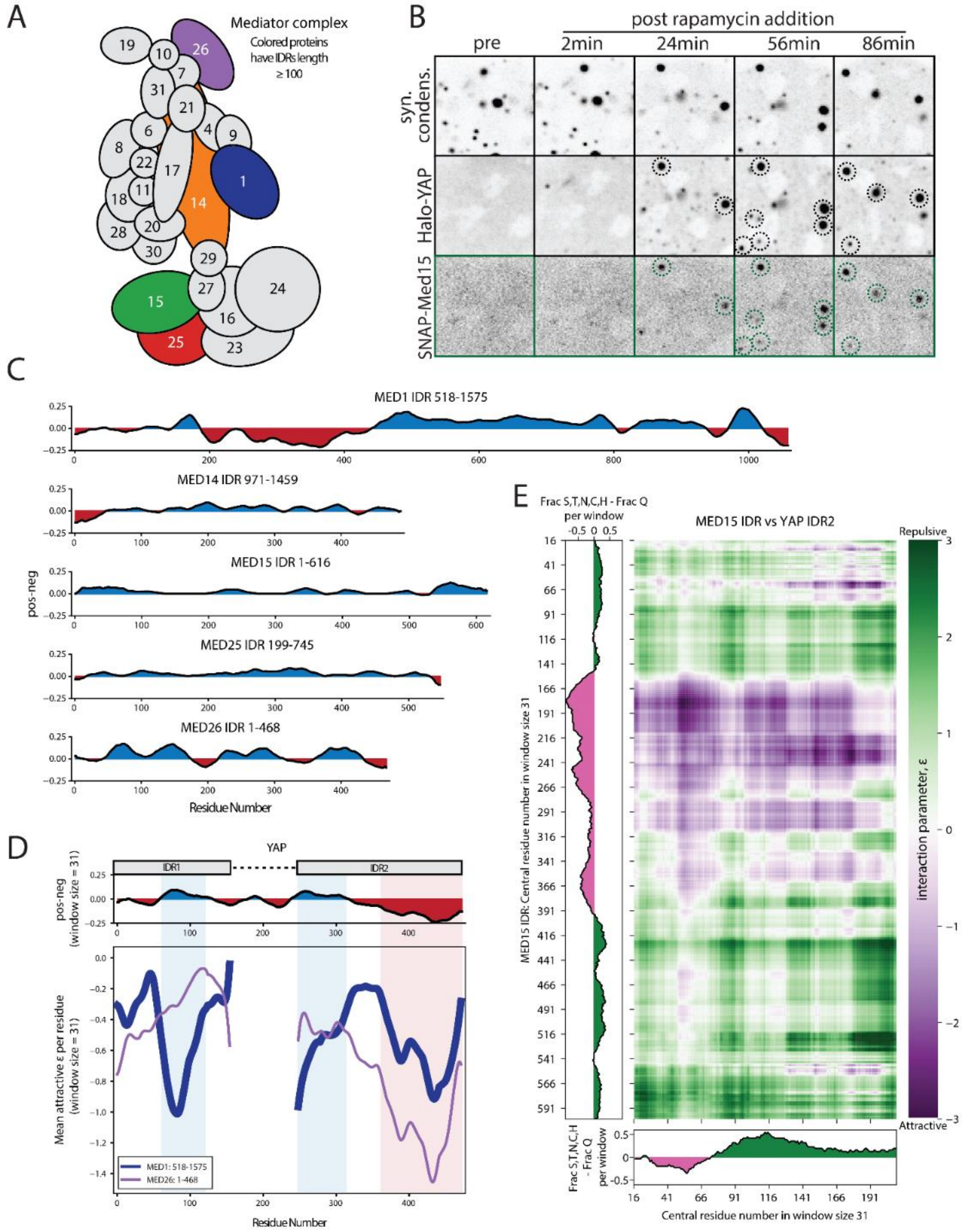

Figure S5 Analysis of intermolecular interactions between YAP and IDR containing Mediator subunits

A) Overview of IDR containing subunits of the Mediator complex according to Richter et al.[1] with Mediator subunit organization as shown in Soutourina et al.[2] and Malik[3]. Color-coded subunits have IDRs with length ≥ 100 residues. **B**) Time series images of acute YAP recruitment (middle row) to pre-formed synthetic condensates (top row) and the resulting response of the endogenous Med15 protein. YAP was recruited to synthetic condensates at 2 min following rapamycin addition. Green and black dashed circles indicate Med15/YAP double positive condensates. **C)** Analysis of charged residues in IDR containing Mediator subunits. The graphs show the mean net charge per residue using a window size of 31 along the sequence of the indicated Mediator subunit IDR. **D**) Prediction of intermolecular attractions between the Med1 IDR (blue line) or Med26 IDR (purple line) and the YAP IDR sequences (aligned on top). The top graph shows the mean net charge profile along the YAP protein sequence, IDRs are indicated in grey. Bottom graph shows the FINCHES predicted mean attractive interaction parameter ε of the Med1 (blue line) or Med26 (purple line) IDR for each position along the YAP IDR sequence[4]. Note the alignment of the precited interactions with the IDR charge patterns of YAP IDRs (Med1 interactions align with charge blocks of both YAP IDRs; Med26 interactions align with YAP IDR2's negative charge block). **E**) Predicted intermolecular interaction maps for the Med15 IDR and YAP IDR2. Attractive (magenta) and repulsive (green) interaction strengths are predicted based on the chemical physics of the Mpipi forcefield. Graphs on the left and bottom show the enrichment of glutamine (pink) and other polar residues (green) along the IDRs of Med15 and YAP. Note the alignment of the precited interactions with the Q-rich regions of Med15 IDR and the glutamine and other polar-rich regions of YAP IDR2 (sum of S,T,N,C,H residues; green).

A

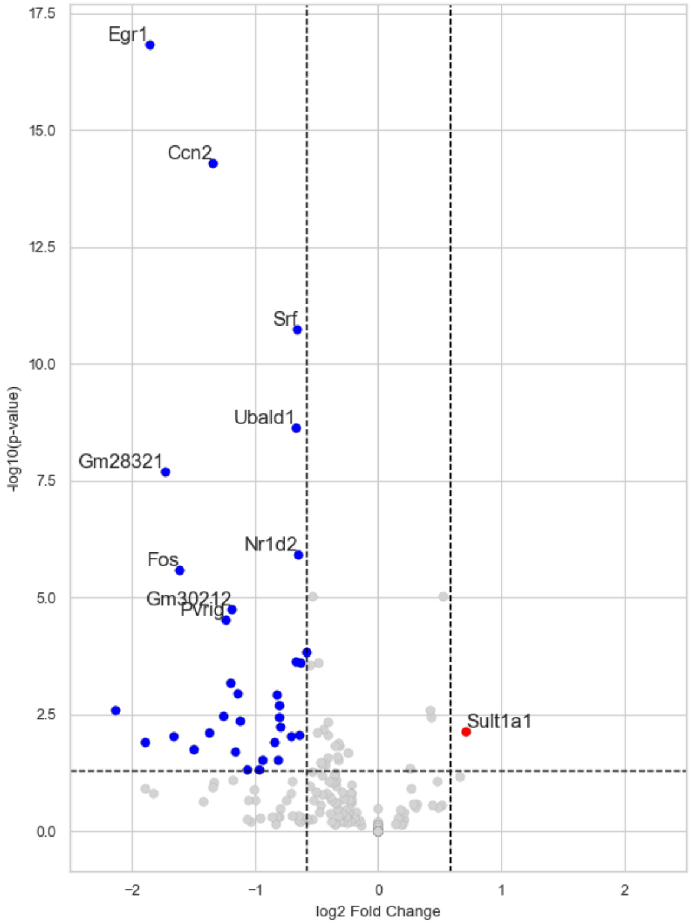

B

Term	Count	P-Value
cAMP signaling pathway	5	0.00069
Human T-cell leukemia virus 1 infection	5	0.001
Pathways in cancer	5	0.017
TNF signaling pathway	4	0.0013
Oxytocin signaling pathway	4	0.0028
Efferocytosis	4	0.0033
Kaposi sarcoma-associated herpesvirus infection	4	0.0079
MAPK signaling pathway	4	0.017
Leishmaniasis	3	0.0075
IL-17 signaling pathway	3	0.013
Neurotrophin signaling pathway	3	0.021
Relaxin signaling pathway	3	0.024
Lipid and atherosclerosis	3	0.062
Salmonella infection	3	0.08

Figure S6 Analysis of gene regulation by synthetic YAP-Med1 co-condensates
A) Volcano plot showing differentially expressed genes regulated by synthetic YAP-Med1 co-condensates. Fold change represents the ratio of gene expression in cells with synthetic condensates at 1 hour post rapamycin-induced YAP recruitment versus cells with synthetic condensates and YAP expression, but without rapamycin addition. Significantly upregulated or downregulated transcripts are shown in red or blue, respectively (|fold change| ≥ 1.5, P-value < 0.05). **B**) KEGG Pathway analysis of differentially regulated genes in cells with rapamycin-induced synthetic YAP condensates. Count indicates the number of differentially expressed genes in a pathway.

Supplementary References

1. Richter, W. F., Nayak, S., Iwasa, J. & Taatjes, D. J. The Mediator complex as a master regulator of transcription by RNA polymerase II. *Nat. Rev. Mol. Cell Biol.* **23**, 732–749 (2022).
2. Soutourina, J. Transcription regulation by the Mediator complex. *Nat. Rev. Mol. Cell Biol.* **19**, 262–274 (2018).
3. Malik, S. Eukaryotic transcription regulation: Getting to the heart of the matter: Commentary on mediator architecture and RNA polymerase II function by plaschka et al. *J. Mol. Biol.* **428**, 2575–2580 (2016).
4. Ginell, G. M., Emenecker, R. J., Lotthammer, J. M., Usher, E. T. & Holehouse, A. S. Direct prediction of intermolecular interactions driven by disordered regions. *bioRxivorg* (2024) doi:10.1101/2024.06.03.597104.
